# Duration of type 2 diabetes mellitus and systolic blood pressure as determinants of severity of coronary stenosis and adverse events in an asymptomatic diabetic population: PROCEED study

**DOI:** 10.1186/s12933-019-0855-8

**Published:** 2019-04-23

**Authors:** Shreenidhi M. Venuraju, Avijit Lahiri, Anand Jeevarethinam, Mark Cohen, Daniel Darko, Devaki Nair, Miranda Rosenthal, Roby D. Rakhit

**Affiliations:** 1 Institute of Cardiovascular Science, University College London, London, UK; 2 British Cardiac Research Trust, 62 Century Court, London, NW8 9LD UK; 3 Bedford Hospital, Bedford, UK; 4 Cardiac Imaging and Research Centre, Wellington Hospital, London, UK; 5 Imperial College School of Medicine, Imperial College London, London, UK; 6 Healthcare Science, Middlesex University, London, UK; 7 Norfolk and Norwich University Hospital, Norwich, UK; 8 Barnet Hospital, Barnet, UK; 9 The Jeffrey Kelson Centre for Diabetes and Endocrinology, Central Middlesex Hospital, London, UK; 10 Department of Diabetes and Endocrinology, Royal Free Hospital, London, UK; 11 Department of Cardiology, Royal Free Hospital, London, UK

**Keywords:** Atherosclerosis, Computed tomography coronary angiography, Coronary artery calcium, Risk stratification, Silent coronary artery disease, Type 2 diabetes mellitus

## Abstract

**Background:**

Evidence from imaging studies suggests a high prevalence of coronary artery disease (CAD) in patients with type 2 diabetes mellitus (T2DM). However, there are no criteria for initiating screening for CAD in this population. The current study investigated whether clinical and demographic characteristics can be used to predict significant CAD in patients with T2DM.

**Methods:**

Computed tomography coronary angiography (CTCA) and laboratory assessments were performed in 259 patients diagnosed with T2DM attending clinics in Northwest London, UK. Coronary artery calcium (CAC) was calculated during CTCA. Significant plaque was defined as one causing more than 50% luminal stenosis. Associations between groups and variables were evaluated using Student’s *t* test, Chi-square tests and univariate and multivariate regression analysis. P < 0.05 was considered statistically significant.

**Results:**

Among patients with a median duration of T2DM of 13 years and a mean age of 62.0 years, median CAC score was 105.91 Agatston Units. In a multivariate analyses, duration of diabetes, CAC score and the presence and number of coronary artery plaques and presence of significant plaque were significant predictors of cardiovascular adverse events. Systolic blood pressure (SBP) had borderline significance as a predictor of cardiovascular events (p = 0.05). In a receiver operating characteristic curve (ROC) analysis, duration of diabetes of > 10.5 years predicted significant CAD (sensitivity, 75.3%; specificity 48.2%). Area under the ROC curve was 0.67 when combining duration of T2DM > 10.5 years and SBP of > 139 mm Hg. Adverse cardiovascular events after a median follow-up of 22.8 months were also significantly higher in those with duration of T2DM > 10.5 years and SBP > 140 mm Hg (log rank p = 0.02 and 0.009, respectively).

**Conclusions:**

Routine screening for CAD using CTCA should be considered for patients with a diagnosis of T2DM for > 10.5 years and SBP > 140 mm Hg.

*Trial registration* Clinicaltrials.gov identifier: NCT02109835, 10 April 2014 (retrospectively registered)

## Background

A diagnosis of type 2 diabetes mellitus (T2DM) doubles the risk of developing coronary artery disease (CAD) compared with controls and leads to accelerated atherosclerosis [1]. Accordingly, approximately one-third of patients with T2DM have cardiovascular (CV) comorbidities, most commonly atherosclerosis (29.1%) and CAD (21.2%) [2]. Furthermore, approximately half of deaths among patients with T2DM are attributed to CV causes, with CAD contributing to the cause of death in approximately 60% of cases [2].

Patients with T2DM and CAD may be asymptomatic because T2DM-related autonomic neuropathy can mask anginal symptoms of CAD, which can act as a warning sign for patients who do not have T2DM [3]. However, there is no clear evidence of a clinical benefit when screening an unselected population of patients with T2DM for CAD, so no universally accepted screening guidelines have been issued.

Various investigative modalities have shown promise as screening tests for establishing a hierarchy of risk. For example, coronary artery calcium (CAC) score can predict long-term CV risk in patients with T2DM [4], but offers an incomplete picture, as evidenced by the higher CV morbidity in patients with T2DM compared with those without T2DM with similar CAC scores [5]. The difference in mortality between patients with and without T2DM may be attributable to a combination of a greater prevalence of non-calcified, and thus more ‘vulnerable’, plaque lesions and various systemic factors, including the pro-inflammatory milieu associated with T2DM.

Patients with T2DM also have a higher myocardial ischaemic burden when examined using myocardial perfusion scintigraphy (MPS) [6]. However, ischaemia had resolved at follow-up in 79% of participants with ischaemia on their initial MPS scan, possibly due to intensified medical management of CV risk factors following the initial scan [6]. Furthermore, ischaemia does not necessarily correlate with epicardial luminal stenosis [7], particularly in patients with T2DM in whom ischaemia on MPS scans could be attributable to microvascular disease or endothelial dysfunction [8].

Computed tomography coronary angiography (CTCA) can be used to evaluate the coronary anatomy, along with the extent and severity of any coronary artery atherosclerosis, providing detailed information regarding the composition of plaque, plaque burden and remodeling of plaque. Observations from CTCA also correlate well with invasive angiography and have a high sensitivity for diagnosing CAD [9].

Retrospective studies have previously demonstrated the high prevalence of coronary plaque in high-risk, but asymptomatic, patients with T2DM and CAD [10], but it is unclear when screening for CAD should be initiated in this patient population. Therefore, this study aimed to evaluate the prevalence of coronary atherosclerosis in asymptomatic subjects with T2DM, and the factors affecting the extent and severity of CAD in these patients, as well as the clinical and demographic factors associated with adverse CV events. In addition, the study aimed to determine the optimal time for initiating screening for CAD in patients with T2DM.

## Methods

This multicentre study recruited patients with T2DM from hospital diabetes clinics and a CV screening clinic in North-West London (Barnet Hospital, Central Middlesex Hospital, Royal Free Hospital and North West London Cardiovascular Screening Community Clinic) as part of the Progression of Coronary Atherosclerosis in Asymptomatic Diabetic Subjects: Evaluation of the Role of CT Coronary Angiography and Markers of Endothelial Function and Vascular Inflammation (PROCEED) study. All patients aged ≥ 35 years who had been diagnosed with T2DM for ≥ 1 year enrolled in these clinics were invited to participate in the study. Pregnant women, patients with known CAD, those with a known, severe allergy to iodinated contrast media and patients with an estimated glomerular filtration rate < 45 mL/min/1.73 m^2^ were excluded. Patients with known atrial fibrillation were also excluded.

All patients included in the study provided written informed consent before enrollment. This study was retrospectively registered with Clinical Trials.gov after the first patient was enrolled in September 2012 (Clinicaltrials.gov identifier: NCT02109835) in concordance with the Seventh Revision of the Declaration of Helsinki (2013). Ethical approval was obtained from National Research Ethics Service, UK.

All patients were screened using a non-contrast CAC scan. Patients with a CAC score > 1000 Agatston units (AU) did not undergo CTCA because they were presumed to have significant coronary stenosis. An adverse CV event was defined as all-cause death, non-fatal myocardial infarction (MI) or late coronary revascularisation. MI was defined as any CV event that associated with a significant increase in cardiac troponin levels associated with symptoms or electrocardiographical changes of myocardial ischaemia. Late coronary revascularisation was defined as any revascularisation procedure that was undertaken more than 60 days after baseline investigations.

### CTCA scan protocol

All scans were acquired using a dual source computed tomography scanner (Somatom Definition, Forchheim, Germany) with a maximum load capacity of 220 kg. The scan protocol consisted of: (1) topogram; (2) prospectively gated, non-enhanced scan to calculate CAC score according to standard protocol using the TeraRecon (Foster City, CA, USA) workstation [11]; (3) test bolus scan to determine the circulation time; and (4) contrast-enhanced coronary angiogram.

Immediately before the scan, 800 µg of sub-lingual nitroglycerin was administered. Intravenous metoprolol (up to 15 mg) was administered, in patients with a heart rate (HR) > 70 beats per minute (bpm), except where β-blockers were contraindicated.

All images were acquired cranio-caudally, in the supine position during a single breath-hold in inspiration. Images were acquired with a gantry rotation time of 330 ms, detector collimation of 2 × 32 × 0.6 mm (with double sampling in the z axis, using flying focal spot technology), pitch of 0.2–0.5 (adapted to HR) with retrospective electrocardiogram (ECG) gating and ECG-controlled tube current modulation. Scan parameters, such as tube current and tube voltage, were altered for each patient based on body mass index (BMI) and truncal adiposity. A triple-phase contrast protocol was used with a bolus of contrast (Iomeron 400, Bracco, Italy) followed by 30 mL of a 30:70 mixture of contrast and saline followed by a 70 mL saline flush, all administered at a rate of 5.5–6 mL/s. Contrast bolus volume (mL) was calculated based on scan time (s) multiplied by flow rate, plus 10 mL (minimum volume: 60 mL).

### Image reconstruction and analysis

CTCA images were reconstructed in mid-diastole for patients with HR < 70 bpm and for end-systole in patients with HR > 70 bpm. If initial reconstructions were not satisfactory, additional reconstructions were made every 5% of the R–R interval and reviewed in an attempt to identify the best quality data set. Images were reconstructed with a slice thickness of 0.75 mm and increment of 0.5 mm using B26 heart view convolution kernel. Images were then transferred to a dedicated workstation (Leonardo, Siemens, Forchheim, Germany). Axial slices, multiplanar reconstructions and maximum intensity projections were used to evaluate the patency of coronary arteries [11].

All images were analysed by two readers (SV and AJ; each with > 4 years’ experience in CTCA). Segmentation was based on the American Heart Association 18-segment model, as recommended by the Society of Cardiovascular Computed Tomography. All contrast opacified vessels with a diameter > 1.5 mm were included in the analysis. Degree of stenosis was assessed as percent reduction in luminal diameter versus reference vessel diameter.

### Safety follow-up

Patients were routinely followed up by telephone or email for adverse CV events. All self-reported adverse CV events were confirmed by review of medical records.

### Statistical analysis

Baseline characteristics were described using frequencies and percentages for categorical variables. For normally distributed continuous variables, mean and standard deviation (SD) are presented. Median and interquartile range (IQR) are presented for data that are not normally distributed. Summaries were produced for all patients, and then separately for the subgroup with CTCA measurements that were used in subsequent analyses.

Univariate logistical regression analysis was performed for both clinically relevant CAD and adverse CV events. The strength of associations was further assessed using multivariate analyses. Covariates for the clinically relevant CAD and adverse CV event analyses were identified as having p < 0.05 and p < 0.20, respectively, in univariate analyses. A backwards selection procedure was used to retain only statistically significant variables in the final adverse CV event model. The multivariate adverse CV event analysis was performed twice; first considering CAC, and subsequently omitting this variable.

Receiver operator characteristic (ROC) curve analysis was used to identify optimal diagnostic sensitivity and specificity. Adverse CV events were assessed as time to event and analysed using the Kaplan–Meier method and a Cox proportional hazards regression model. Patients who did not report an adverse CV event were censored at the time of last follow-up. The log-rank test was used for comparing the equality of survival distributions.

P < 0.05 was considered statistically significant for all analyses. Any variable with more than 15% missing data was excluded from the final analysis. All data was analysed using STATA (StataCorp LLC, College Station, TX, USA).

## Results

A total of 259 patients with a mean (SD) age of 61.6 (8.5) years were enrolled. Overall, 55.6% of subjects were male and 57.6% were of South Asian origin, reflecting the ethnic composition of the screening population and high prevalence of T2DM in this ethnic group. Mean duration (SD) of T2DM was 13.7 (7.8) years. A full description of the baseline characteristics of the study population are presented in Table 1.

**Table 1:** Baseline characteristics of the PROCEED study population

	Overall population, (n = 259)	Subgroup with CTCA data, (n = 238)
Age, mean years (SD)	62.0 (8.5)	61.6 (8.6)
Sex, male (%)	151 (59)	133 (56)
BMI, median kg/m^2^ (IQR)	28.4 (25.3, 32.5)	28.6 (25.5, 32.6)
Ethnicity, n (%)		
African	34 (13)	33 (14)
South Asian	142 (56)	127 (54)
Caucasian	79 (31)	75 (32)
Duration of diabetes, median years (IQR)	13 (8, 19)	13 (8, 19)
Microvascular disease, n (%)	123 (48)	114 (48)
Retinopathy, n (%)	99 (38)	90 (38)
Hyperlipidaemia, n (%)	203 (78)	187 (79)
Hypertension, n (%)	192 (74)	176 (74)
Smoking, n (%)	20 (8)	19 (8)
Statin use, n (%)	188 (73)	173 (73)
Family history of premature ischaemic heart disease, n (%)	51 (20)	45 (19)
SBP, mm Hg (SD)	137.3 (15.8)	137.1 (15.7)
HbA_1c_, median mmol/mol (IQR)	63.0 (51.9, 77.0)	64.0 (51.9, 77.0)
eGFR, mean mL/min/1.73 m^2^ (SD)	80.9 ± 18.4	80.9 ± 18.8
CAC (AU), median (IQR)	109 (1, 322)	82 (0, 266)
Total cholesterol, mean mmol/L (SD)	4.05 (0.92)	4.08 (0.93)
LDL-C, mean mmol/L (SD)	2.10 (0.88)	2.12 (0.89)
HDL-C, mean mmol/L (SD)	1.29 (0.39)	1.29 (0.39)
Triglycerides, median mmol/L (IQR)	1.4 (0.9, 1.9)	1.4 (1.0, 1.9)
Total cholesterol:HDL-C ratio, mean (SD)	3.41 (1.2)	2.41 (1.2)
Medications for diabetes, n (%)		
Metformin	232 (90)	214 (90)
Sulphonylureas	87 (34)	78 (33)
Thiazolidinediones	21 (8)	18 (8)
GLP-1 agonists	36 (14)	34 (14)
DPP-4 Inhibitors	50 (19)	47 (20)
Insulin	139 (54)	132 (55)
ACEi/ARB	163 (63)	149 (62)

ACEi, angiotensin-converting enzyme inhibitor; ARB, angiotensin receptor blocker; AU, Agatston Units; BMI, body mass index; CAC, coronary artery calcium; CAD, coronary artery disease; eGFR, estimated glomerular filtration rate; HbA_1c_, glycated haemoglobin; HDL-C, high-density lipoprotein cholesterol; IQR, interquartile range; LDL-C, low-density lipoprotein cholesterol; SD standard deviation

### Extent of coronary calcification

Mean (SD) CAC score was 334.48 (651.4) AU. A CAC score of zero was observed in 62 (24%) subjects, while 64 (24.7%) subjects had mild coronary calcification (CAC score < 100 AU). Severe coronary calcification (CAC score > 400 AU) was observed in 57 (22%) patients, of which 20 (7.7%) had a CAC score > 1000 AU and were referred back to their diabetologist for further management without undergoing CTCA. More than 15% of data was missing for one patient, who did not proceed to CTCA and excluded from further analysis.

### Factors determining prevalence of significant stenosis

Of the 238 subjects who underwent a CTCA, 94 (39.5%) had ≥ 1 coronary plaque causing > 50% luminal diameter stenosis (clinically relevant CAD). Patients with clinically relevant CAD had a significantly longer duration of diabetes, higher systolic blood pressure (SBP), lower serum high-density lipoprotein cholesterol (HDL-C) levels, and a higher prevalence of microvascular disease (Table 2).

**Table 2:** Predictors of a clinically relevant CAD identified using logistical regression analysis

Univariate predictors	Univariate analysis		Multivariate analysis	
	OR (95% CI)	p value	OR (95% CI)	p value
Age (per 10 years)	1.39 (1.02–1.91)	*0.04*	0.99 (0.96–1.04)	0.99
Male sex	1.85 (1.08–3.16)	*0.02*	2.09 (1.07–4.08)	*0.03*
BMI (per 5 kg/m^2^)	0.77 (0.62–0.96)	*0.02*	0.95 (0.90–1.00)	0.054
Waist-to-hip ratio (per unit increase)	1.13 (0.80–1.59)	0.49		
Ethnicity		*0.03*		*0.002*
South Asian	1		1	
Caucasian	0.51 (0.28–0.93)		0.43 (0.21–0.89)	
African	0.35 (0.15–0.83)		0.18 (0.06–0.51)	
Duration of T2DM (per 5 years)	1.38 (1.15–1.63)	< *0.001*	1.59 (1.27–1.98)	< *0.001*
Microvascular disease (Yes/no)	1.87 (1.10–3.16)	*0.02*	0.71 (0.33–1.5)	0.36
Retinopathy (Yes/no)	1.62 (0.95– 2.76)	0.08		
Hyperlipidaemia (Yes/no)	1.26 (0.66– 2.39)	0.49		
Hypertension (Yes/no)	3.63 (1.81–7.29)	< *0.001*	0.68 (0.28–1.64)	0.39
Current smoker (Yes/no)	1.12 (0.43– 2.91)	0.80		
Statin use (Yes/no)	1.06 (0.59– 1.91)	0.84		
Family history of premature ischaemic heart disease (Yes/no)	0.89 (0.46– 1.75)	0.74		
SBP (per 10 mm Hg)	1.43 (1.19–1.72)	< *0.001*	1.37 (1.09–1.72)	*0.007*
HbA_1c_ (per 10 mmol/mol)	1.10 (0.94–1.27)	0.24		
eGFR (per 10 mL/min/1.73 m^2^)	0.89 (0.77– 1.03)	0.12		
CAC score (per log_10 _AU)	3.79 (2.60–5.53)	< *0.001*		
Total cholesterol (per mmol/L)	1.21 (0.92– 1.60)	0.18		
LDL-C (per mmol/L)	1.27 (0.94– 1.70)	0.12		
HDL-C (per mmol/L)	0.40 (0.19–0.83)	*0.01*	0.95 (0.29–3.13)	0.93
Triglycerides (per mmol/L)	1.15 (0.90– 1.48)	0.27		
Total cholesterol:HDL-C ratio (per unit)	1.41 (1.12–1.77)	*0.003*	1.84 (1.37–2.46)	< *0.001*
Antidiabetic medication (Yes/no)				
Metformin	1.24 (0.54– 2.86)	0.61	9.93 (1.91–51.62)	*0.006*
Sulphonylurea	0.83 (0.48–1.44)	0.51		
Thiazolidinedione	5.7 (1.28–25.42)	*0.02*		
GLP-1 agonist	1.42 (0.66–3.07)	0.37		
DPP-4 inhibitor	1.49 (0.76–2.93)	0.25		
Insulin	1.16 (0.69–1.96)	0.58		
ACEis/ARBs (Yes/no)	0.53 (0.30–0.91)	*0.02*	1.80 (0.93–3.50)	0.08

CAD is defined as ≥ 1 coronary plaque causing > 50% luminal diameter stenosis

Italics indicate a statistically significant association between the predictor variable and clinically relevant CAD

ACEi, angiotensin-converting enzyme inhibitor; ARB, angiotensin receptor blocker; AU, Agatston Units; BMI, body mass index; CAC, coronary artery calcium; CAD, coronary artery disease; CI, confidence interval; DPP-4, dipeptidyl peptidase-4; GLP-1 glucagon-like peptide-1; HDL-C, high-density lipoprotein cholesterol; LDL-C low-density lipoprotein cholesterol; OR, odds ratio; SBP, systolic blood pressure; T2DM, type 2 diabetes mellitus

Univariate regression identified age, male gender, BMI (inverse association), ethnicity, microvascular disease, duration of diabetes, SBP, HDL-C, total cholesterol:HDL-C ratio, thiazolidinedione therapy, angiotensin-converting enzyme inhibitor/angiotensin receptor blocker therapy (inverse association) and CAC score as predictors of stenosis. In a multivariate analysis, only gender, ethnicity, duration of T2DM, SBP, total cholesterol:HDL-C, thiazolidinedione therapy remained predictors of significant plaque. Log transformed CAC score was a significant predictor (OR: 4.85, 95% CI 2.95–7.99, p < 0.001) when added to the above model and all the other variables also retained significance except for gender (p = 0.38).

### Optimal cut-off points for duration of diabetes and SBP

Area under the ROC curve analysis for the presence of clinically relevant CAD was 0.64 for duration of diabetes, 0.65 for SBP and 0.82 for CAC score. Cut-off points at 10.5 years and 12.5 years of T2DM were identified as offering a high sensitivity for predicting a clinically relevant CAD with reasonable specificity. An SBP cut-off of 131 mm Hg and 139 mm Hg offered similar performance (Table 3). The area under the ROC for a combination of duration of T2DM of 12.5 years and SBP of 131 mm Hg was 0.70, and 0.67 for a combination of duration of T2DM of 10.5 years and SBP of 139 mm Hg, but these values were lower than for CAC score alone.

**Table 3:** Sensitivity and specificity for various significant predictor variables identified using the ROC coordinates

Variable cut-offs	Sensitivity, %	Specificity, %
Duration of T2DM—10.5 years	75.3	48.2
Duration of T2DM—12.5 years	71.0	56.1
SBP—131 mm Hg	75.3	50.4
SBP—139 mm Hg	59.1	59.0

ROC, receiver–operator curve; SBP, systolic blood pressure; T2DM, type 2 diabetes mellitus

### Adverse cardiovascular events

Safety follow-up was performed for 250 subjects over a median of 22.8 months. Nine subjects were lost to follow-up. In total, 18 adverse CV events were noted, including six deaths, one ischaemic stroke and 11 late revascularisations (seven percutaneous coronary interventions and four coronary artery bypass graft surgeries).

In a univariate Cox regression model, age, waist-to-hip ratio, duration of T2DM, SBP, CAC score and plaque variables (number of plaques and presence of significant plaque) were predictors of adverse CV events (Table 4). Following a multivariate Cox regression analysis, only duration of T2DM, CAC score, total number of plaques and the presence of significant plaque were associated with an increased probability of an adverse CV event (Table 5). SBP was borderline significant for predicting adverse CV events (p = 0.05).

**Table 4:** Factors associated with adverse CV events in a univariate Cox regression analysis

Variable	Hazard ratio (95% CI)	p value
Age (per 10 years)	2.45 (1.37–4.37)	*0.003*
Male sex	3.50 (1.01–12.1)	0.05
BMI (per 5 kg/m^2^)	0.83 (0.55–1.27)	0.40
Waist-to-hip ratio (per unit increase)	2.04 (1.06–3.92)	*0.03*
Ethnicity		0.31
South Asian	1	
Caucasian	1.64 (0.63–4.26)	
African	0.45 (0.06–3.52)	
Duration of T2DM (per 5 years)	1.36 (1.05–1.75)	*0.02*
Microvascular disease (Yes/no)	1.14 (0.45–2.88)	0.78
Retinopathy (Yes/no)	0.84 (0.32–2.25)	0.74
Hyperlipidaemia (Yes/no)	0.53 (0.20–1.40)	0.20
Hypertension (Yes/no)	1.21 (0.40–3.66)	0.74
Current smoker (Yes/no)	0.67 (0.09–5.03)	0.70
Statin use (Yes/no)	0.57 (0.23–1.48)	0.25
Family history of premature ischaemic heart disease (Yes/no)	0.75 (0.22–2.59)	0.65
SBP (per 10 mm Hg)	1.43 (1.11–1.84)	*0.006*
HbA_1c_ (per 10 mmol/mol)	1.10 (0.86–1.41)	0.44
eGFR (per 10 mL/min/1.73 m^2^)	0.82 (0.63–1.08)	0.17
CAC score(per log_10_ AU)	3.17 (1.60–6.29)	*0.001*
Total cholesterol (per 10 mmol/L)	0.90 (0.53–1.51)	0.69
LDL-C (per 10 mmol/L)	1.06 (0.63–1.81)	0.82
HDL-C (per 10 mmol/L)	0.47 (0.12–1.77)	0.26
Triglycerides (per 10 mmol/L)	1.03 (0.69–1.54)	0.89
Total cholesterol: HDL-C ratio	1.33 (0.91–1.95)	0.13
Antidiabetic medication		
Metformin	0.76 (0.09–5.88)	0.76
Sulphonylurea	0.96 (0.29–3.18)	0.94
Thiazolidinedione	22.74 (0.002–245,100)	0.51
GLP-1 agonists	1.835 (0.24–14.21)	0.56
DPP-4 inhibitors	2.67 (0.35–20.71)	0.35
Insulin	1.30 (0.50–3.36)	0.58
ACEis/ARB (Yes/no)	0.6 (0.19–1.86)	0.38
Plaque variables^a^		
Number of plaques	1.28 (1.13–1.45)	< *0.001*
Presence of 50% plaque	8.09 (1.80–36.91)	*0.007*
Number of 50% plaques	1.32 (1.18–1.46)	< *0.001*

Italics indicate a statistically significant association between the predictor variable and adverse CV events

ACEi, angiotensin-converting enzyme inhibitor; ARB, angiotensin receptor blocker; AU, Agatston Units; BMI, body mass index; CAC, coronary artery calcium; CI, confidence interval; CV, cardiovascular; DPP-4, dipeptidyl peptidase-4; GLP-1 glucagon-like peptide-1; HDL-C, high-density lipoprotein cholesterol; LDL-C low-density lipoprotein cholesterol; SBP, systolic blood pressure; T2DM, type 2 diabetes mellitus

^a^Analysis excludes the 20 patients with CAC score > 1000 who did not undergo CTCA

**Table 5:** Factors associated with adverse CV eventsin a multivariate Cox regression analysis

Model	**N**	Variable	Hazard ratio (95% CI)	p value
1^a^	250	Duration of T2DM (per 5 years)	1.35 (1.05–1.74)	*0.02*
		CAC score (per log_10_ AU)	2.32 (1.12–4.81)	*0.02*
2^b^	234	Number of 50% plaques^c^	1.17 (1.01–1.36)	*0.04*
		Number of plaques^c^	1.19 (1.03–1.37)	*0.02*
		SBP (per 10 mm Hg)	1.04 (1.0–1.08)	0.05

Italics indicate a statistically significant association between the predictor variable and adverse CV events

AU, Agatston units; CAC, coronary artery calcium; CI, confidence interval; CV, cardiovascular; SBP, systolic blood pressure; T2DM, type 2 diabetes mellitus

^a^Patients with CAC score data who were available for safety follow-up

^b^Patients with CTCA data who were available for safety follow-up

^c^Analysis excludes the 20 patients with CAC score > 1000 who did not undergo CTCA

### Survival analysis

**Fig. 1:**
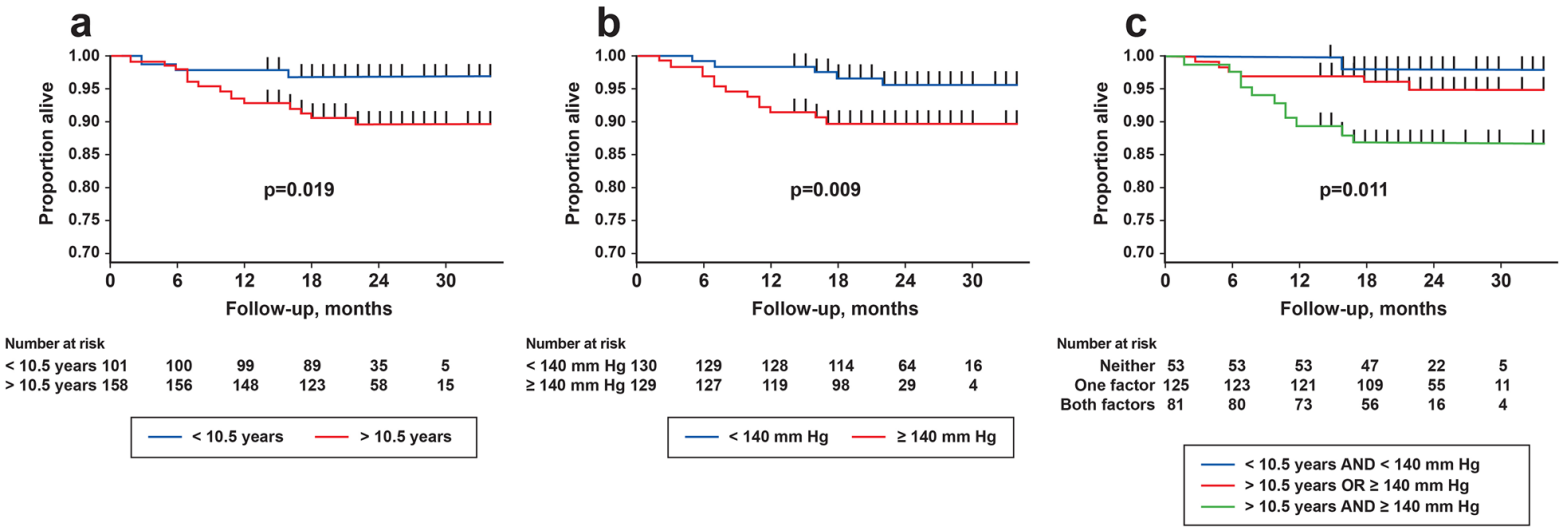
Adverse CV events survival probability by **a** duration of T2DM, **b** SBP, and **c** combined score. Kaplan–Meier adverse CV event survival analysis during the follow-up period (median follow-up: 22.8 months). Cumulative score (ranging from 0 to 2) was based on duration of diabetes > 10.5 years and systolic blood pressure of 140 mm Hg. CV, cardiovascular; SBP, systolic blood pressure

When duration of T2DM was treated as a binary variable, with 10.5 years and 12.5 years as cut-offs, only the 10.5-year cut-off was significantly associated with the probability of an adverse CV event (p = 0.019 and p = 0.163 for 10.5- and 12.5-year cut-offs, respectively; Fig. 1a). Similarly, SBP cut-offs of 131 mm Hg (p = 0.041) and 140 mm Hg (p = 0.009) were significantly associated with adverse CV events (Fig. 1b).

We hypothesised that combining duration of diabetes and SBP into one variable, providing a cumulative score, would offer a significant advantage in predicting survival. A score of 0 was attributed to subjects with a duration of T2DM < 10.5 years and SBP < 140 mm Hg (rounded for ease of application); a score of 1 was attributed to those with either a duration of diabetes > 10.5 years or SBP > 140 mm Hg and a score of 2 given to those with a duration of T2DM > 10.5 years and SBP > 140 mm Hg. Using this scoring system, there was a clear separation of curves for the different scores (p = 0.001) (Fig. 1c).

## Discussion

There is considerable interest in identifying clinical and demographic factors that influence the prevalence of CAD in patients with T2DM because of the high CV morbidity and mortality in this population. Occult CAD in this population is not just limited to coronary atherosclerosis, but also to myocardial ischaemia, given that > 20% of asymptomatic patients with T2DM have experienced a silent MI [6]. However, no difference in the rate of cardiac death and non-fatal MI was observed between patients randomised to myocardial perfusion versus no screening [6], although anatomical and functional tests, in series, may be used to complement one another by utilising CAC imaging as the initial `gatekeeper’ test [4]. Earlier results from the PROCEED study indicated that endothelial function does not predict CV events in patients with T2DM [12], but CAC progression occurs faster in patients with T2DM versus patients with no or pre-diabetes who are symptomatic for CAD [13]. Accordingly, CAC score has been suggested as a potential predictor of CAD and future adverse CV events. However, optimal timing for initiating screening in patients with T2DM has not yet been elucidated.

### Screening for CAD in patients with T2DM

CTCA offers an alternative to invasive catheter angiography for screening for CAD and has very high sensitivity (95–99%) [14]. Notably, in this study CTCA identified a high prevalence (nearly 40%) of occult CAD in an asymptomatic sample of patients with T2DM, which is consistent with other studies reporting a prevalence of CAD in patients with T2DM ranging from 64 to 80%, including significant, obstructive plaques being observed in 26–37% of subjects [15–18]. In contrast, < 10% of a general population may be expected to present with similar plaques [18, 19]. Furthermore, the prevalence of CAD in this study was observed in the context of a population that was optimally treated for hypertension and dyslipidaemia, with mean values being well within the recommended national guidelines [20].

However, despite the high prevalence of CAD patients with T2DM, there is limited prognostic data to support screening asymptomatic patients because no significant difference in CV outcomes have been reported between groups screened with CTCA versus routine care, especially when other CV risk factors, such as hypertension and dyslipidaemia are well-managed [6, 21]. However, given the prognostic significance of the extent and severity of CAD, as assessed by CTCA [22], this study attempted to identify clinical and demographic factors that predict the presence of clinically relevant CAD, defined as plaque causing > 50% luminal stenosis. Age, male gender, BMI, duration of T2DM, presence of hypertension, SBP and HDL-C were noted to be univariate predictors of clinically relevant CAD, but in a multivariate analysis, only duration of T2DM and SBP were predictors of clinically relevant CAD, while serum HDL-C levels offered negative predictive value. These factors remained significant, independent of CAC score.

### Identifying candidates for routine screening for CAD

Duration of T2DM (> 10.5 years) and SBP (> 139 mm Hg) were also the only clinical and demographic factors that remained significantly associated with adverse CV events following multivariate analysis, which is consistent with the British Regional Heart study that indicated that a duration of T2DM of > 10 years increases the risk of an adverse CV event to an equivalent level of a patient with CAD, independently of the presence of clinically relevant CAD [23]. Therefore, these factors were incorporated into a simple scoring system, assigning duration of diabetes and SBP with a score of 1 or 0 based on the optimal cut-off points identified by ROC curve analyses. Subsequently, all patients were assigned a score of 0, 1 or 2. Kaplan–Meier analysis shows a good separation of the curves for the three distinct scores with a p value of < 0.001. Hence, patients who have been diagnosed with T2DM for > 10.5 years and with SBP > 140 mm Hg were found to have a higher risk of presenting with clinically relevant CAD and were also more likely to suffer from an adverse CV event.

Therefore, patients with a score of 2 using this system (i.e., a duration of T2DM > 10.5 years and SBP > 140 mm Hg) should be considered for CAD screening with CTCA. Patients with clinically relevant CAD could then be aggressively treated with antihypertensive and low-density lipoprotein cholesterol–lowering therapies. However, a larger, more diverse validation cohort randomised into screening and no screening populations, and with a longer duration of follow-up, would be required to confirm a clinical benefit from screening patients for CAD using this algorithm (i.e., reduced CV morbidity and mortality following screening).

### Study limitations

This study is limited by its relatively small sample size, restricted geographic nature and study population that is limited to patients with T2DM receiving care in a secondary setting. In addition, duration of T2DM was calculated from the time a diagnosis was documented in patients’ clinical notes. Therefore, periods when patients may have displayed impaired glucose tolerance prior to starting antidiabetic treatment have not been accounted for. Likewise, single measurements of glycated haemoglobin only provided a snapshot of near-term glycaemic and blood pressure levels, and did not assess the relevance of long-term glycaemic and blood pressure control on the risk of developing CAD, although this could be considered to be reflective of normal clinical practice. A mean duration of safety follow-up of < 2 years is also a relatively short timeframe within which to observe major CV events. As a result, a low number of events were recorded, limiting the statistical power of the safety analysis.

## Conclusions

A combined duration of T2DM of > 10.5 years and SBP ≥ 140 mm Hg is significantly associated with an elevated risk of asymptomatic CAD and adverse CV events in patients with T2DM. These two clinical factors are also predictors of significant coronary stenosis and can be used as a guide to initiate CV screening in patients with T2DM.

## Abbreviations

ACEi: angiotensin-converting enzyme inhibitor
ARB: angiotensin receptor blocker
AU: Agatston units
bpm: beats per minute
BMI: body mass index
CAC: coronary artery calcium
CAD: coronary artery disease
CTCA: computed tomography coronary angiography
CV: cardiovascular
DPP-4: dipeptidyl peptidase 4
ECG: electrocardiogram
GLP-1: glucagon-like peptide 1
HDL-C: high-density lipoprotein cholesterol
HR: heart rate
IQR: inter-quartile range
LDL-C: low-density lipoprotein cholesterol
MI: myocardial infarction
MPS: myocardial perfusion scintigraphy
PROCEED: Progression of Coronary Atherosclerosis in Asymptomatic Diabetic Subjects: Evaluation of the Role of CT Coronary Angiography and Markers of Endothelial Function and Vascular Inflammation
ROC: receiver operating characteristic
SBP: systolic blood pressure
SD: standard deviation
T2DM: type 2 diabetes mellitus
